# Semaphorin 7A is protective during inflammatory peritonitis through integrin receptor signaling

**DOI:** 10.3389/fimmu.2023.1251026

**Published:** 2023-11-29

**Authors:** Andreas Körner, David Köhler, Mariella Schneider, Judith M. Roth, Tiago F. Granja, Claudia Eggstein, Valbona Mirakaj, Peter Rosenberger

**Affiliations:** ^1^ Department of Anesthesiology and Intensive Care Medicine, University Hospital, Tübingen, Germany; ^2^ Department of Obstetrics and Gynecology, Augsburg University Hospital, Augsburg, Germany; ^3^ CBIOS-Universidade Research Center for Biosciences & Health Technologies, Universidade Lusófona, Lisbon, Portugal

**Keywords:** SEMA7A, inflammation, peritonitis, integrin, neuronal guidance cues

## Abstract

**Introduction:**

The study explores the role of endothelial Semaphorin 7A (SEMA7A) in inflammatory processes. SEMA7A is known for enhancing inflammation during tissue hypoxia and exhibiting anti-inflammatory properties in the intestinal system during colitis. This research extends the understanding of SEMA7A's function by examining its role in inflammatory peritonitis and intestinal inflammation.

**Methods:**

The research involved inducing peritonitis in SEMA7A knockout (*SEMA7A^-/-^*) and wild-type (WT) animals through Zymosan A (ZyA) injection. The inflammatory response was assessed by measuring cell count and cytokine release. In parallel, the study investigated the expression of SEMA7A in intestinal epithelial cells under inflammatory stimuli and its impact on interleukin 10 (IL-10) production using an *in vitro* co-culture model of monocytes and epithelial cells. Additionally, the distribution of SEMA7A target receptors, particularly ITGAV/ITGB1 (CD51/CD29), was analyzed in WT animals.

**Results:**

The results revealed that *SEMA7A^-/-^* animals exhibited increased inflammatory peritonitis compared to the WT animals. Inflammatory conditions in intestinal epithelial cells led to the induction of SEMA7A. The co-culture experiments demonstrated that SEMA7A induced IL-10 production, which depended on integrin receptors and was independent of PLXNC1 expression. Furthermore, ITGAV/ITGB1 emerged as the predominant SEMA7A receptor in the intestinal area of WT animals.

**Discussion:**

These findings underscore the multifaceted role of SEMA7A in inflammatory processes. The differential responses in peritonitis and intestinal inflammation suggest that SEMA7A's function is significantly influenced by the expression and distribution of its target receptors within different organ systems. The study highlights the complex and context-dependent nature of SEMA7A in mediating inflammatory responses.

## Introduction

Control of inflammation is a complex process that is governed by the interplay of leukocytes, cytokines, chemokines, and tissue-derived factors. Recent work has indicated that acute inflammation is not only influenced by the classical system of cytokines but also by the system of neuronal guidance proteins. This work has shown that parallels exist between the nervous system and the immune system (1, 2). For example, activating signals that induce the growth cone of axons during embryonic development can also activate the migration of leukocytes. Vice versa, netrin-1 stops the axonal growth cone from spreading and also stops leukocytes from migrating into inflammatory tissue sites (3, 4).

Recently, we demonstrated that SEMA7A, a guidance protein belonging to the semaphorin family, plays a crucial role in inducing acute inflammation and promoting the migration of neutrophils to hypoxic tissue sites (5). Semaphorins are a diverse group of proteins, both secreted and present on cell surfaces, known to regulate neurite extension. SEMA7A specifically triggers the production of cytokines in macrophages and monocytes (6–8). Moreover, SEMA7A also promotes cytoskeletal reorganization in melanocytes and monocytes, resulting in changes in cell morphology, such as spreading and migration (9–11). Recent studies have also suggested that SEMA7A could play a potential part in triggering inflammation in cases of lung injury induced by seawater aspiration (12) or following lipopolysaccharide challenge (13) and contribute to metabolic reprogramming in peritoneal macrophages (14). In a recent study, we were also able to show that PNCs (platelet–neutrophil complexes) are increased in patients with acute myocardial infarction and this is associated with increased levels of SEMA7A (15, 16). However, controversial data exist for the role of SEMA7A in the intestinal system, since a study conducted by Kang et al. reported that SEMA7A demonstrated a protective role during DSS-induced colitis (17).

SEMA7A carries out its various functions by interacting with specific receptors, such as ITGA1, ITGAV, and ITGB1. These receptors play a key role in cell adhesion and migration. Moreover, SEMA7A also binds to Plexin C1, which is involved in immune regulation. Gaining an understanding of the intricate interplay between SEMA7A and these receptors is essential for comprehending its function in both normal physiological processes and disease states, including its impact on cytokine regulation. We aimed here to extend our knowledge about the role of SEMA7A in the immune system and to continue our previous work on the role of SEMA7A during hypoxia. For this, we induced Zymosan A (ZyA) peritonitis in WT and *SEMA7A^-/-^* animals. We did this on the background of the conflicting data about the role of SEMA7A during colitis and other entities to generate further evidence about the role of SEMA7A during inflammation.

## Materials and methods

### Experimental animals

The animal procedures followed the guidelines for the ethical use of living animals in Germany and received approval from both the Institutional Animal Care and Use Committee of Tübingen University Hospital and the Regierungspräsidium Tübingen, Germany. Semaphorin 7A knockout (*SEMA7A^-/-^*) mice (obtained from The Jackson Laboratory, Maine, USA) and their littermate controls were selected based on matching criteria including sex, age, and weight.

### CaCo-2 cell line

CaCo-2 cells have been widely utilized as a model of the intestinal barrier due to their origin in human colon adenocarcinoma tissues and were obtained from ATCC. Upon reaching confluence, CaCo-2 cells spontaneously differentiate, acquiring several morphological and functional characteristics of mature enterocytes. For this, cells were grown for an additional period of 15-21 days to fully differentiate. Cells were grown in Eagle’s Minimum Essential Medium supplemented with 20% fetal bovine serum and 1% penicillin-streptomycin at 37°C in a humidified atmosphere of 5% CO2.

### Murine model of ZyA-induced peritonitis

*SEMA7A^-/-^* and WT were injected i.p with either 1ml of PBS or 1ml PBS containing ZyA (ZyA, 1mg/ml, (Sigma-Aldrich, Cat. Nr. Z4250-1G)) to induce peritonitis. After 4 hours from the injection peritoneal lavage and organs were harvested for further analysis. All reagents used were endotoxin-free.

### Transcriptional analysis

CaCo-2 cells were grown to confluency and stimulated with 100ng/ml TNF-α, 20ng/ml IL-6, or 20ng/ml IL-1β in a time-dependent manner. Human SEMA7A transcript was detected by real-time PCR, using the following primer sets: sense primer 5’- CTC AGC ATC CAG CGA CAT -3’ and the antisense primer 5’- ACA GGG GCA CTA TCC ACA AG -3’. qPCR analysis of murine SEMA7A transcript was conducted using the sense primer 5’- GTG GGT ATG GGC TGC TTT TT-3’ and antisense primer 5’- CGT GTA TTC GCT TGG TGA CAT –3’. For the organ screening of WT and *SEMA7A^-/-^*, the following primer sets were used: ITGB1 (CD29), sense primer 5’- GGT GTC GTG TTT GTG AAT GC-3’ and antisense primer 5’- TCC TGT GCA CAC GTG TCT T -3’, ITGA1 (CD49a), sense primer 5’- CCT TCC CTC GGA TGT GAG TCA -3’ and antisense primer 5’- AAG TTC TCC CCG TAT GGT AAG A -3’, ITGAV (CD51), sense primer 5’- CAA GCT CAC TCC CAT CAC -3’ and antisense primer 5’- GGG TGT CTT GAT TCT CAA AGG G -3’ and PLXNC1, sense primer 5’- TTA GGA AGG AGG CGA AGA GA -3’ and antisense primer 5’- ACA GAG ACG CCA ATG ACA AG -3’. For analysis of the SEMA7A receptors in CaCo-2 cells, the following primer sets were used: ITGB1, sense primer 5’- GAA GGG TTG CCC TCC AGA -3’ and antisense primer 5’- GCT TGA GCT TCT CTG CTG TT -3’, ITGA1, sense primer 5’- GGT TCC TAC TTT GGC AGT ATT -3’ and antisense primer 5’- AAC CTT GTC TGA TTG AGA GCA -3’, ITGAV sense primer 5’- AAT CTT CCA ATT GAG GAT ATC AC -3’ and antisense primer 5’- AAA ACA GCC AGT AGC AAC AAT -3’ and PLXNC1, sense primer 5’- TTG TCA TTT TTG CGG CCG TG -3’ and antisense primer 5’- GGG TGA AGC CAC CTG ACT C -3’. Samples for CaCo-2 and murine tissue were controlled using 18S as housekeeping gene (sense primer 5’- GTA ACC CGT TGA ACC CCA TT -3’ and antisense primer 5’- CCA TCC AAT CGG TAG TAG CG -3’).

### MPO and protein measurement in peritoneal lavage

For Myeloperoxidase (MPO) measurement, 50µl of peritoneal lavage was used. Then, 50 µl citrate buffer and 100 µl ABTS solution were added to the samples, and samples were incubated light-protected for 25 minutes at 37°C. To determine the protein levels, measurements were performed in accordance with the standard protocol of the BCA Protein Assay Kit (Thermo Scientific; Cat. Nr. 23225). Measurements were performed on an infiniteM200PRO TECAN Reader at 405 nm (for MPO) and 540 nm (for protein).

### Protein analysis

After homogenization, the isolated protein samples from CaCo-2 cells or murine colon tissue were resuspended in RIPA buffer. The protein levels were determined as described above. The samples were normalized for protein levels and loaded to 10% SDS polyacrylamide gels. After blotting on a PVDF membrane, a polyclonal rabbit anti-SEMA7A antibody (Abcam, Cat. Nr. ab23578) was used for detection in both cell and murine tissue samples. Polyclonal rabbit GAPDH (Santa Cruz; Cat. Nr. sc-25778) antibody was used to control loading conditions. A goat anti-rabbit IgG antibody conjugated with horseradish peroxidase (HRP) was employed for detection (Santa Cruz, sc-2004). Bands were detected by using Western Blotting Luminol Reagent (Santa Cruz, Cat. Nr. sc 2048). Densitometric analysis was performed normalizing SEMA7A protein levels to GAPDH using Image J 1.44p.

### Cell counts

For cell counts, 10µl of the peritoneal lavage that was harvested after 4 hours of either ZyA or PBS injection was diluted in 10 ml of CASY ton solution (Roche, Cat. Nr.: 05 651 808 001) and analyzed using the CASY Model TT (Roche Diagnostics GmbH, Mannheim, Germany) cell counter.

### Measurement of peritoneal lavage cytokines

All cytokine concentrations in peritoneal lavage were measured using standard ELISA protocols following the manufacturer’s instructions. ELISAs were performed for TNF-α, IL-6, IL-1β, KC, and MIP-2 (R&D Systems, Minneapolis, USA).

### Semaphorin 7A ELISA

SEMA7A ELISAs were conducted according to manufacturers’ instructions (ELISA Kit Uscn, Life Science Inc.; Kit for SEMA7A human; Cat. Nr. SEB448Hu and for SEMA7A murine; Cat. Nr. SEB448Mu). *In vitro*, 100µl supernatant was removed from CaCo-2 cells after stimulation with TNF-α (100ng/ml) for 24h. For negative control, the supernatant of vehicle-stimulated cells was used. In animal experiments, blood and peritoneal lavage were analyzed after i.p. injection of 1mg/ml ZyA and 4 hours incubation time.

### Semaphorin 7A reporter assay

For analyzing the promoter region and to identify prospective NF-κB-binding sites, search engines such as MatInspector and BioBase were used. Vector pGL4.17-expressing sequence corresponding to full-length SEMA7A promoter – either native (SEMA7A FL, -1041 to +29, 1.069 bp) or containing mutations in one of the following potential NF-κB binding sites (NF-κB1 (bp -1000 to -1008), NF-κB2 (bp -818 to -826), NF-κB3 (bp -675 to -683), and NF-κB4 (bp -188 to -196)) – were employed from GeneArt (Regensburg, Germany). For the negative control, the empty Vector pGL4.17 was used. For positive control, the pNF-κB-Luc (pNRE) from Clontech (Palo Alto, CA, USA) was purchased. Precluding that new NF-κB-binding sites were generated, the new sequences were controlled by using the abovementioned search engines. Transfection of cells was performed according to standard conditions of PolyFect Transfection Reagent (QIAGEN, Cat. Nr. 301105). Following incubation with 100ng/ml TNF-α for 24h the luciferase activity was determined by employing a Steady-Glo Luciferase Assay System (Promega, Madison, USA). Luciferase was normalized concerning the total protein amount.

### Immunofluorescence staining

CaCo-2 cells were grown on chamber slides (Thermo Scientific, Cat. Nr. 177399) and stimulated for 4 hours as described previously. Next, CaCo-2 cells were washed with PBS, fixed, and permeabilized for 10 minutes in acetone and methanol 1:1. After being washed with PBS, they were blocked with 5% BSA in PBS for 1 hour and stained with a polyclonal goat anti-human Semaphorin 7A Antibody (R&D Systems, Cat. Nr. AF2068). For the secondary antibody, polyclonal donkey anti-goat Alexa Fluor 488 was used (Thermo Scientific, Cat. Nr. A-11055). Nuclei were counterstained with DAPI (Carl Roth GmbH, Cat. Nr. HP 20.1). β-Actin staining was performed using a monoclonal rabbit β-Actin antibody (13E5) (Cell Signaling, Cat. Nr. 4970S). To visualize β-Actin, a polyclonal goat anti-rabbit Alexa Fluor 594 (Thermo Scientific, Cat. Nr. A-11012) was used as the secondary antibody.

For murine immunofluorescence stainings, colons were harvested, embedded in TissueTek, and snap-frozen. Sections were washed three times in PBS and blocked with 5% BSA in PBS for 1 hour. Polyclonal rabbit Anti-Semaphorin 7a antibody (Abcam, Cat. Nr. ab23578) served as the detection antibody. A polyclonal goat anti-rabbit Alexa Fluor 488 (Thermo Scientific, Cat. Nr. A-11008) was used as the secondary antibody. Nuclei counterstains were performed as described above. For structural staining, a polyclonal goat antibody Cytokeratin 12 (L-15) (Santa Cruz, Cat. Nr. sc-17101) was used. A polyclonal donkey anti-goat Alexa Flour 549 (Thermo Scientific, Cat. Nr. A-11058) was used as the secondary antibody. Fluorescent imaging of murine and cell samples was performed with an Axiophot Zeiss microscope (Zeiss, Germany) using a digital camera with the AxioVision 4.8 software.

### Immunohistologic staining

For murine histological staining, peritoneal tissue was cryopreserved and fixed in methanol. PMN staining of peritoneal tissues was employed by using Vectastain ABC Kit (Linaris, Mannheim Germany). For blocking, avidin blocking solution (Vector Labs, Burlingame, USA) was used and incubated for 1 hour at room temperature. For PMN staining, the sections were incubated overnight at 4°C at a dilution of 1:1000 with a monoclonal rat Ly-6B.2 alloantigen antibody 7/4 (AbD Serotec, Cat. Nr. MCA771GA) or rat IgG control (Santa Cruz, Cat. Nr. sc-2026). After incubation of the tissue sections with biotinylated rabbit anti-rat IgG (Vector Labs, Cat. Nr. BA-4000) for 30 minutes, the sections were incubated with Vectastain ABC Reagent (Vector Labs, Cat. Nr. PK-4000) for 30 minutes. For the development of the tissue stainings, Histogreen (Linaris, Cat. Nr. E109) was used as a substrate. Nuclear fast red (Linaris, Cat. Nr. H-3403) was employed for counterstaining. Examination of the tissue sections was performed under a Leitz DM IRB microscope (Leica). Analysis was performed using the AxioVision v4.8.2 software.

### Stimulation and interaction of MM6 and CaCo-2 cells

For intestinal co-culture, CaCo-2 cells were grown to confluency and joined with 5×10^6^ MM6 cells. Then they were stimulated with 50µg/ml ZyA (Sigma Aldrich, Cat. Nr. Z4250) only or in combination with 100ng/ml recombinant human Semaphorin 7A Fc Chimera Protein (R&D Systems, Cat. Nr. 2068-S7). After incubation for 4 hours, 100µl supernatant was harvested and analyzed to determine IL-10 following the manufacturer’s instructions via ELISA (R&D Systems, Cat. Nr. DY217B). In a subset of experiments, CaCo-2 and MM6 cells were preincubated for 1 hour at room temperature with one of the following antibodies: 1:500 ITGA1 (Anti-Integrin alpha 1 antibody, (Abcam, Cat. Nr. ab33410)) or 1:1000 ITGAV/ITGB1 (Rabbit Anti-Integrin Alpha V + Beta1 Polyclonal Antibody (Bioss, Cat. Nr. bs-2016R)), 1:1000 ITGB1 (Anti-Integrin β1; abcam, Cat Nr. ab52971) or 1:500 Anti-PLXNC1 (Santa Cruz, Cat. Nr. Sc10149), respectively, before adding ZyA and/or recombinant human Semaphorin 7A Fc Chimera Protein. 

### *In vitro* TNFα and hypoxia stimulation protocol

CaCo-2 and A549 cells were counted and seeded in 6-well plates. For hypoxia experiments, the cells were directly seeded in pre-prepared hypoxic media and exposed to conditions of 2% oxygen for 24 hours. In TNFα stimulation experiments, the cells were treated with TNFα at a concentration of 100 ng/ml for 24 hours. Following incubation, RNA extraction was performed by adding Trizol directly to each well followed by further steps as per the instructions provided by the manufacturer.

### Data analysis

Statistical significance was assessed using one-way ANOVA, followed by Bonferroni’s, Dunnett’s and Holm-Šídák's multiple comparison tests when needed. The Student’s t-test was utilized where appropriate. A p-value below 0.05 was considered statistically significant.

## Results

### Semaphorin 7A is protective in inflammatory peritonitis

**Figure 1:**
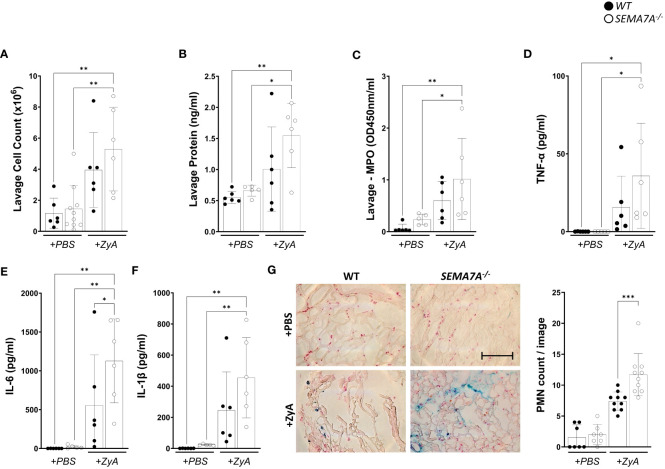
*SEMA7A^-/-^* mice show increased severe inflammation during induced peritonitis. Peritonitis was induced in WT and *SEMA7A^-/-^* animals through intraperitoneal injection of either phosphate-buffered saline (PBS) or Zymosan A (ZyA). The degree of inflammation was determined 4 hours following injection. Depiction of **(A)** Cell counts **(B)** protein levels **(C)** MPO activity in the peritoneal lavage of WT and *SEMA7A^-/-^* mice. **(D)** TNF-α, **(E)** IL-6, and **(F)** IL-1β (pg/ml) concentration were measured in the peritoneal lavage of WT and *SEMA7A^-/-^* animals. Additional measurements for KC and MIP-2 are shown in **Supplementary Figures 1A, B**. **(G)** Staining and counting of PMNs in histological sections from the peritoneum of WT and *SEMA7A^-/-^* animals treated with either ZyA or PBS. One representative image of peritoneal tissue out of three different animals per group is depicted (scale bar 100 μm). For negative controls see **Supplementary Figure 2**. (data are mean ± SD, *P < 0.05; **P < 0.01, ***P < 0.001 as indicated, n≥5/group, **(G)** n=3 (PBS treatment) 7 pictures/group evaluated, n=3 (ZyA treatment) 10 pictures/group evaluated).

We first tested whether SEMA7A holds pro-inflammatory actions during inflammatory peritonitis and as such holds similar properties as we have described before. To our great surprise, this was not the case and we found that *SEMA7A^-/-^* mice demonstrated pro-inflammatory behavior compared to littermate controls. This finding was corroborated by measuring the cell count, protein content, and myeloperoxidase activity within the peritoneal lavage (**Figures 1A–C**). In addition, the concentration of pro-inflammatory cytokines TNF-α, IL-6, IL-1β (**Figures 1D–F**), KC (CXCL1) and MIP-2 (CXCL2) (**Supplementary Figure 1**) within the peritoneal lavage also demonstrated that SEMA7A holds protective potential during peritonitis. The anti-inflammatory effect of SEMA7A in peritonitis was reflected in PMN sequestration into the peritoneum where *SEMA7A^-/-^* mice were intensively affected compared to WT mice (**Figure 1G**; **Supplementary Figure 2**).

### SEMA7A is induced *in vivo* during peritonitis

**Figure 2:**
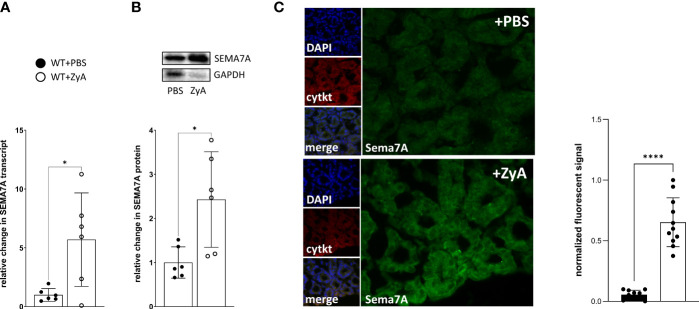
Induction of SEMA7A *in vivo* during ZyA-induced peritonitis. Colon tissues from WT animals were removed 4 hours after intraperitoneal administration of either PBS or ZyA. **(A)** RT-PCR analysis of colon tissue demonstrates the relative change in SEMA7A mRNA following either PBS or ZyA injection. **(B)** Demonstration of Western Blot as well as the densitometric analysis of SEMA7A protein expression in the colon tissue following ZyA exposure and **(C)** Immunofluorescence analysis of SEMA7A (green) in the colon tissue (n=3/group). Cytokeratin (cytkt/red) served as an epithelial marker to show tissue structure. Negative controls are displayed in **Supplementary Figure 3**. (**A** and **B** n=6/group, all data are mean ± SD, *P < 0.05, ****P < 0.0001 as indicated).

Next, we quantified whether SEMA7A is induced or repressed during peritonitis. For this, we used harvested murine tissues from *SEMA7A^+/+^* -mice and performed RT-PCR and protein analysis. Following exposure to ZyA, we observed the upregulation of SEMA7A in colon tissues as evidenced by transcriptional and protein analysis (**Figures 2A, B**; **Supplementary Figure 7**). Immunofluorescent labeling of SEMA7A showed an induced fluorescent signal for SEMA7A in the colon of WT animals exposed to ZyA (**Figure 2C**; **Supplementary Figure 3**).

### Regulation of SEMA7A induction *in vitro* by pro-inflammatory cytokines via NF-κB transcription factor

**Figure 3:**
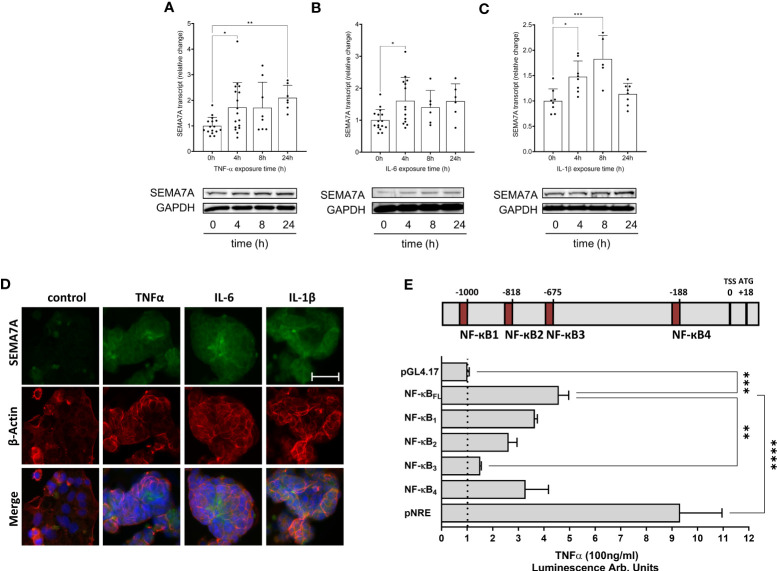
Expression of SEMA7A in CaCo-2 during inflammation. Human intestinal CaCo-2 cell lines were stimulated with pro-inflammatory cytokines. RT-PCR and Western Blot analysis of SEMA7A-mRNA and protein expression after stimulation of CaCo-2 cells with **(A)** 100ng/ml TNF-α **(B)** 20ng/ml IL-6 and **(C)** 20ng/ml IL-1β for indicated periods. **(D)** SEMA7A immunofluorescence staining of intestinal CaCo-2 cells following 4 hours of exposure to TNF-α, IL-6, and IL-1β (scale bar 50 μm). For negative controls see **Supplementary Figure 5**. **(E)** Depiction of potential NF-κB binding sites in the SEMA7A promoter region. Relative to the transcription start site (TSS) in the upstream sequence of SEMA7A, four potential binding elements of NF-κB were determined. CaCo-2 cells were transfected with the SEMA7A reporter plasmid, containing either the native NF-κB full length (NF-κB_FL_) or site-directed mutations in one of the binding sites for NF-κB. As negative control empty pGL4.17 plasmid was used, pNF-κB-Luc (pNRE) was used for positive control (all data are mean ± SD, *P < 0.05; **P < 0.01; ***P < 0.001, ****P < 0.0001 as indicated, n=6-17/group, one representative Western Blot and one representative IF image were selected).

Next, we tested whether the induction of SEMA7A could also be observed *in vitro*. For this, we used intestinal epithelial CaCo-2 cells grown to confluence and exposed these cells to the pro-inflammatory cytokines after incubation for an additional period of 15-21 days. Through analysis at both the transcriptional and protein levels, we observed the induction of SEMA7A following exposure of CaCo-2 to TNF-α, IL-6, or IL-1β (**Figures 3A–C**; **Supplementary Figure 4**). Upon performing immunofluorescent labeling of SEMA7A, we confirmed the presence of an induced fluorescent signal for SEMA7A in CaCo-2 cells in response to TNF-α, IL-6, and IL-1β (**Figure 3D**; **Supplementary Figure 5**). To gain specific insights into the mechanism underlying SEMA7A regulation during inflammation, we analyzed available public databases (18) and identified the transcription start site of human SEMA7A at position +18 relative to the first codon (**Figure 3E**). We further identified four putative binding sites for NF-κB within the SEMA7A promoter region, located at positions -1000bp, -818bp, -675bp, and -188bp relative to the transcription start site. To investigate the functional significance of these binding sites, we utilized luciferase reporter constructs containing the putative full-length SEMA7A promoter (NF-κB_FL_, from TSS to -1041). We also performed site-directed mutagenesis in each single reporter construct to exclude the influence of each NF-κB binding site. The FL construct demonstrated an induction of SEMA7A luciferase activity following incubation with TNF-α. This response could be gradually reduced through elimination of the binding sites NF-κB_1_, NF-κB_2,_ and NF-κB_4_ and significantly reduced through elimination of the binding site NF-κB_3,_ which exerts the main effect (**Figure 3E**).

### CaCo-2 cell-derived SEMA7A undergoes cleavage, which subsequently triggers the transmigration of neutrophils

**Figure 4:**
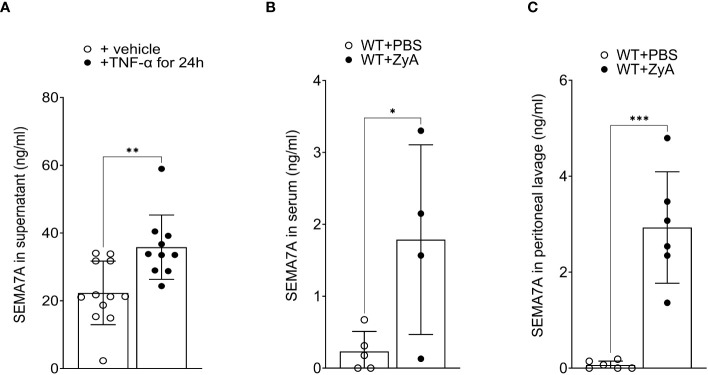
Non-tissue-bound SEMA7A is induced *in vitro* and *in vivo* during inflammation. **(A)** CaCo-2 cells were exposed to 100ng/ml TNF-α for 24 hours and SEMA7A levels were determined in the supernatant following this stimulation. **(B)** Serum concentration of SEMA7A and **(C)** SEMA7A concentration in the peritoneal lavage of WT animals subjected to intraperitoneal administration of either PBS or ZyA (all data are mean ± SD, *P < 0.05; **P < 0.01; ***P < 0.001 as indicated, n=4-12/group).

To find out whether SEMA7A is not only induced during inflammation but also present in a non-tissue-bound form, we then exposed CaCo-2 cells to TNF-α for 24 hours. Following this, we extracted the supernatant and tested for the presence of SEMA7A through ELISA and found a significant increase of SEMA7A in the cell culture supernatant (**Figure 4A**). To pursue this finding further we then evaluated the serum of animals that were exposed to ZyA-induced peritonitis. Here, we also found a significant increase in serum SEMA7A levels following the induction of peritonitis (**Figure 4B**). We then also wanted to know whether SEMA7A is present in the peritoneal cavity of ZyA-exposed mice. When determining this through ELISA we also found a significant induction of SEMA7A levels in the lavage of the peritoneal cavity (**Figure 4C**).

### SEMA7A induces the production of IL-10 through integrin receptors

**Figure 5:**
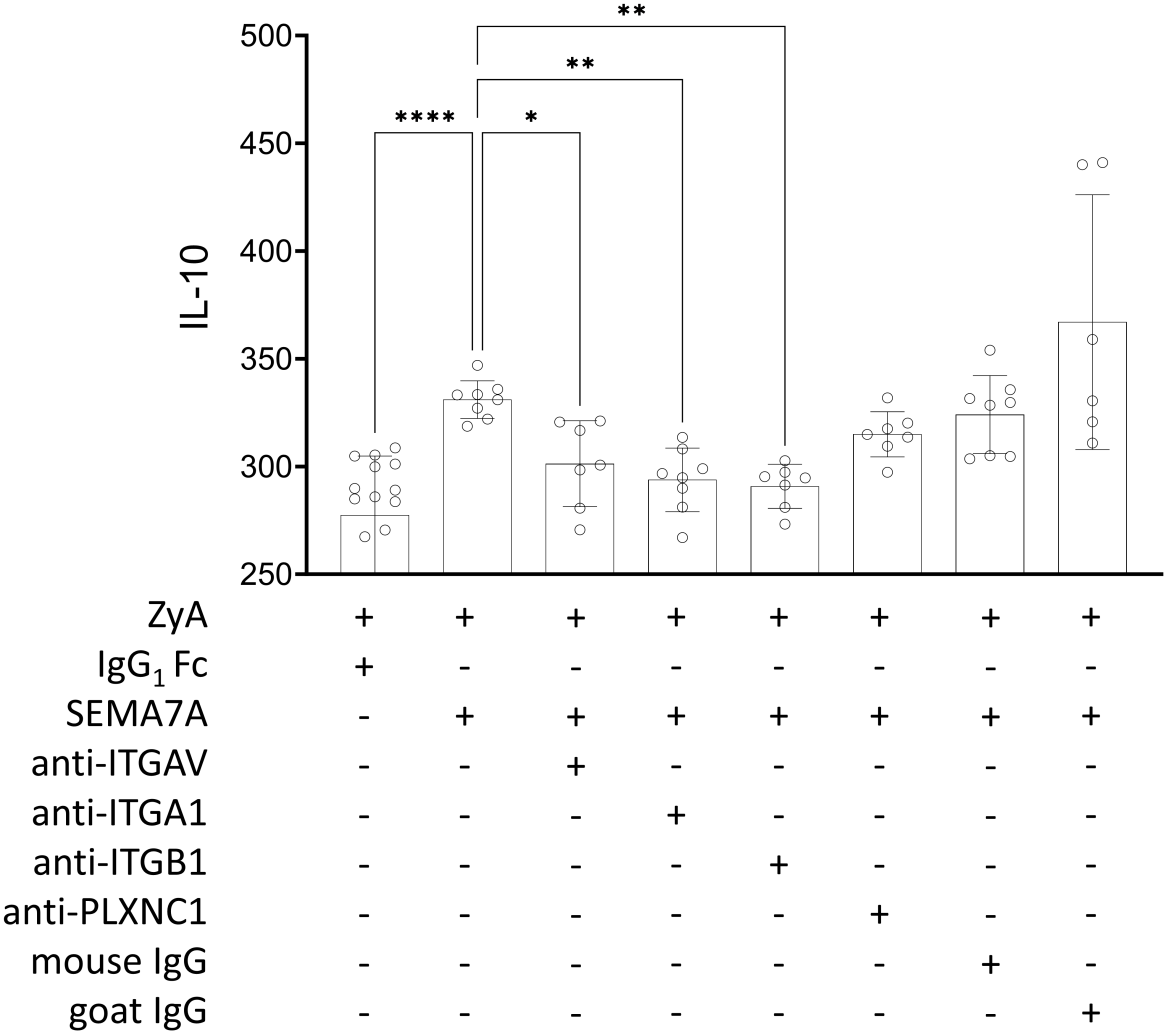
Regulation of IL-10 release induced by rhSEMA7A during inflammation. The effect of rhSEMA7A on IL-10 release was tested in a co-culture of MM6 and CaCo-2 during a ZyA challenge. IL-10 was determined using ELISA, and co-cultured MM6 and CaCo-2 cells were exposed to 50µg/ml ZyA and 100ng/ml rhSEMA7A, respectively. For evaluating the influence of the specific target receptors, the co-culture of CaCo-2 and MM6 cells was pre-incubated for 1 hour with anti-ITGV/ITGB1 integrin antibody, anti-ITGA1 integrin antibody, ITGB1 integrin antibody, anti-PLXNC1 antibody, or IgG control antibody (all data are mean ± SD, *P < 0.05, **P < 0.01 and ****P < 0.0001 as indicated, n≥6/group).

We next decided to test a possible explanation for the above-described findings. For this, we co-cultured MM6 monocytes with intestinal epithelial cell line CaCo-2 and stimulated this setting with recombinant SEMA7A. We found a significant increase in IL-10 production following this. When pre-incubating this setting with an anti-ITGAV antibody, we observed a significant reduction in IL-10 production. We also found this when using anti-ITGA1 and anti-ITGB1 antibodies before exposure to SEMA7A. In addition, once again we did not find an increase in protective IL-10 (**Figure 5**). An increase in IL-10 expression after treatment with recombinant SEMA7A was additionally proven by RT-PCR analysis (**Supplementary Figures 6**, **9**). When using an anti-PLXNC1 antibody we found no significant decrease in IL-10 production. This confirmed the findings described in a previous study (17).

### Expression of SEMA7A target receptors is altered during inflammation

**Figure 6:**
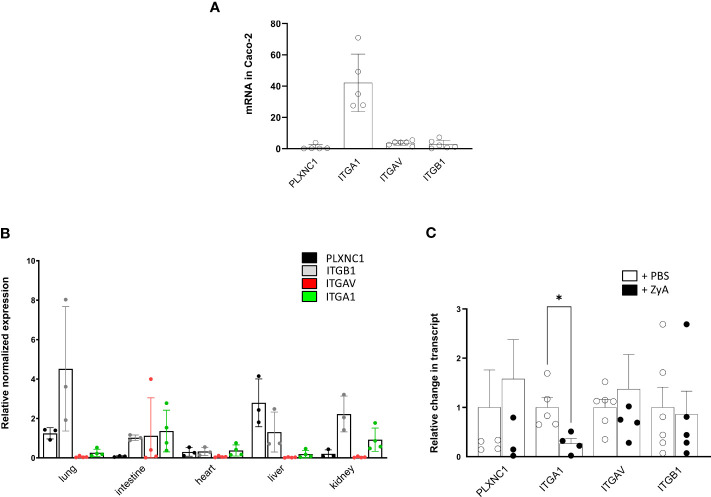
Influence of inflammation *in vitro* and *in vivo* on SEMA7A target receptor expression. **(A)** Depiction of normalized PLXNC1, ITGB1, ITGBV, and ITGA1 integrin receptor expression pattern on CaCo-2 cells **(B)** Murine organ expression patterns of the potential SEMA7A receptors PLXNC1, ITGB1 integrin, ITGAV integrin and ITGA1 integrin in WT animals **(C)** Peritoneal tissue from WT mice were harvested after administration of either PBS or ZyA. Displayed is the relative change in mRNA transcript of PLXNC1, ITGB1 integrin, ITGAV integrin, and ITGA1 integrin mRNA (all data are mean ± SD, *P < 0.05 as indicated, n≥3/group).

Since we were not initially expecting this result when we started these experiments, we then turned our attention to the expression of the potential SEMA7A target receptors. We screened the used CaCo-2 cells and found a prominent expression of ITGA1 and slightly higher expressions of ITGAV and ITGB1 compared to the PLXNC1 receptor expression (**Figure 6A**). We then wanted to evaluate what the expression patterns of these receptors were within the different organs of WT mice. We found that the ITGAV and the ITGA1 receptors were predominantly expressed in the intestine and kidney, and only sparsely in the liver. No expression was found for these receptors within the lung and only rarely in the heart. The ITGB1 integrin receptor was expressed in significant amounts in most organ systems. The PLXNC1 receptor was expressed within the lung and liver, but not in the intestine and kidney (**Figure 6B**). In a final attempt to come closer to an explanation of the described results, we focused on the expression of SEMA7A target receptors in the peritoneum during ZyA-induced peritonitis. We found, reflecting the *in vitro* results from CaCo-2 cells, only significant repression of the ITGA1 integrin receptor, but not of the ITGAV and the ITGB1 integrin receptor. Expression of PLXNC1 was stable in the peritoneal cavity (**Figure 6C**). To investigate the differential expression of target receptors for SEMA7A, we subjected A549 and CaCo-2 cells to varying environmental stimuli, namely, hypoxia and TNFα exposure. Our analyses revealed a divergent expression profile of ITGA1 when the cells were subjected to hypoxic conditions. Additionally, stimulation with TNFα elicited a differential expression pattern not only for ITGA1 but also for ITGAV and ITGB1 across alveolar and intestinal epithelial cells. These observations indicate that the expression of these integrins is subject to complex regulation that is dependent on the tissue origin of the epithelial cells (**Supplementary Figures 8**, **9**).

## Discussion

We have shown in previous research that SEMA7A holds the potential to influence PMN migration during hypoxia (5). We have now extended this work and evaluated which role SEMA7A would play during inflammatory peritonitis. We report here that contrary to our findings during hypoxia, SEMA7A holds significant anti-inflammatory potential during ZyA-induced peritonitis. This confirms previous data from Kang et al. showing that SEMA7A is protective during DSS-induced colitis. Therefore, the protection observed in this context may be attributed to the expression of the target receptors for SEMA7A within the affected organs. As such, SEMA7A seems to hold varying functions depending on the expression of its target receptors.

An increased expression of SEMA7A during pathologic tissue conditions was demonstrated before by us and other researchers. At first, we were able to show this during tissue hypoxia and found that SEMA7A induced on endothelial cells likely engages with the PLXNC1 receptor on neutrophils to induce the migration into hypoxic tissue sites (5). The upregulation of SEMA7A expression on endothelial cells was validated in a study conducted by Zhang et al., employing a lung injury model induced by seawater aspiration (12). In this study, the authors showed the induction of SEMA7A results in an enhancement of lung injury in their model but did not describe a possible target receptor of SEMA7A during their experiments. In previous research, we elucidated that the induction of SEMA7A is via hypoxia-inducible factor 1α (HIF-1α). In the present study, we provide evidence suggesting that SEMA7A is additionally induced through NF-κB during episodes of acute inflammation utilizing CaCo-2 cells as a model system, which is in line with our previous findings during pulmonary inflammation (13). Zhang et al. also identified HIF-1α to be responsible for the SEMA7A induction (12). Earlier studies have established a strong connection between HIF-1α and NF-κB, suggesting the potential induction of SEMA7A through both transcription factors (19). Consequently, it is plausible that SEMA7A is induced via HIF-1α and NF-κB during tissue inflammation, particularly considering the likelihood of inflammatory tissue hypoxia accompanying extensive tissue inflammation (20).

Contrary to our research during hypoxia, we found that SEMA7A does not enhance neutrophil migration and acute inflammation. We found here that SEMA7A demonstrates a protective role in the presented model of ZyA-induced peritonitis. Although we were surprised about this finding, it is in line with previously published data (17). Kang et al. demonstrated that SEMA7A is protective during DSS-induced colitis and produces IL-10 through ITGAV/ITGB1 integrin receptor. We have tested this and can confirm this finding in our model, using a co-culture approach of CaCo-2 intestinal epithelial cells and monocytes. In previous research, we focused on the interaction of SEMA7A with PLXNC1 on neutrophils. However, when evaluating the local environment during ZyA-induced peritonitis we only found very low expression of PLXNC1 on the intestinal organs (for details, see **Figure 6**). Semaphorin 7A mediates its function on axon outgrowth in the central nervous system through ITGB1 integrin (9). During T-cell mediated inflammation, SEMA7A was shown to mediate its effect through ITGA1/ITGB1 integrin receptor and, during pulmonary fibrosis, through ITGB1 integrin (7, 21). We and others have shown that parts of the SEMA7A structure fit into the PLXNC1 receptor binding site and that this mediates, at least in part, the SEMA7A effect. In the present study, we found that SEMA7A is protective and demonstrated that this may be dependent on the receptor expressed in the affected organs. Consistent with the data reported by Kang et al. we also describe the role of the ITGAV/ITGB1receptor in the production of IL-10 and anti-inflammation during intestinal inflammatory changes. Our data evaluating the expression of SEMA7A target receptors support the conclusion that SEMA7A-induced IL-10 production is dependent on the integrin receptors since these receptors are expressed more prominently in the intestinal system than PLXNC1 (**Supplementary Figure 9**). However, these data also demonstrate that the structure of SEMA7A holds the potential to bind to several target receptors.

Our results indicate that SEMA7A receptor targets, including ITGA1, ITGB1, and ITGAV, have various tissue-specific expression patterns. We observed distinct regulation of mRNA in A549 and CaCo-2 cells when exposed to hypoxia or TNFα stimulation. These findings suggest that the role of SEMA7A in mediating inflammation and cytokine production may depend on the specific integrin receptors expressed in different tissues and cell types. It is important to note that our measurements primarily focused on mRNA levels, so further studies are needed to confirm protein expression and determine functional relevance.

In summary, our findings reinforce the critical role of SEMA7A in managing acute inflammatory reactions. Depending on the target receptors expressed within the target tissues, SEMA7A can hold varying functions. We and others have previously shown the pro-inflammatory capacity of SEMA7A. In the present study, we show the anti-inflammatory role of SEMA7A within the peritoneal tissues. Further investigation into the role of SEMA7A and its function through its target receptors is therefore warranted.

## Data availability statement

The raw data supporting the conclusions of this article will be made available by the authors, without undue reservation.

## Ethics statement

Ethical approval was not required for the studies on humans in accordance with the local legislation and institutional requirements because only commercially available established cell lines were used. The animal study was approved by Institutional Review Board of Eberhard Karls Universität Tübingen and Regierungspräsidium Tübingen. The study was conducted in accordance with the local legislation and institutional requirements.

## Author contributions

AK performed experiments, analyzed data, wrote parts of the manuscript; DK performed experiments, analyzed data, wrote parts of the manuscript; MS performed experiments, analyzed data; JR performed experiments, analyzed data; TG performed experiments, analyzed data; CE performed experiments, analyzed data; VM analyzed data, wrote parts of the manuscript; PR designed overall research plan, wrote the manuscript. All authors contributed to the article and approved the submitted version.

## Supplementary material

Supplementary Figure 1 Inflammation markers in the peritoneal lavage of WT and *SEMA7A^-/-^* animals. Peritonitis was induced in WT and *SEMA7A^-/-^* animals through intraperitoneal injection of ZyA. In the control group, PBS-administration was used. The degree of inflammation was determined after 4 hours of incubation by measuring **(A)** KC and **(B)** MIP-2 levels (pg/ml) in the removed peritoneal lavage (all data are mean ± SD, **P* < 0.05; ***P* < 0.01 as indicated, n=5-6/group).

Supplementary Figure 2 IgG and negative controls of PMN staining in histological sections of peritoneum in WT animals after intraperitoneal injection of ZyA (scale bar 100 μm).

Supplementary Figure 3 Depiction of IgG and negative controls for immunofluorescence staining **(A)** SEMA7A and **(B)** Cytokeratin of colon tissue in WT animals (scale bar 50 μm).

Supplementary Figure 4 Expression of SEMA7A in CaCo-2 through cytokines is dose-dependent. Human intestinal CaCo-2 cells were stimulated with increasing doses of pro-inflammatory cytokines. RT-PCR analysis of SEMA7A-mRNA following **(A)** increasing concentrations of TNF-α, **(B)** increasing concentrations of IL-6, and **(C)** increasing concentrations of IL-1β and incubation for 4 hours (All data are mean ± SD, **P* < 0.05; ***P* < 0.01; *****P* < 0.0001 as indicated, n=6-15/group).

Supplementary Figure 5 Demonstration of the IgG and negative controls of CaCo-2 cell culture staining for **(A)** SEMA7A and **(B)** ß-Actin (scale bar 50 μm).

Supplementary Figure 6 Effect of recombinant SEMA7A on the mRNA expression level of IL-10. A co-culture of CaCo-2 and MM6 cells was treated with ZyA (50µg/ml) and SEMA7A (100ng/ml) or ZyA only for 4 hours. The IL-10 expression level was measured by RT-PCR analysis (All data are mean ± SD, ***P* < 0.01 as indicated, n≥6/group).

Supplementary Figure 7 Validation of antibody-specificity. Western Blot of Intestinal tissue from Sema7A WT and SEMA7A^-/-^ mice with SEMA7A antibody **(A)** and densitometric quantification **(B)** (All data are mean ± SD, ***P* < 0.01 as indicated, n=3/group).

Supplementary Figure 8 mRNA expression levels of A549 and CaCo2-cells upon stimulation with hypoxia or TNFα. CaCo-2 and A549 cells were stimulated with either hypoxia **(A)** or **(B)** TNFα and mRNA expression levels of ITGA1, ITGAV, ITGB1, and PLXNC1 were measured by RT-PCR analysis (All data are mean ± SD, ns = not significant *P<0.05, ***P* < 0.01, ****P<0,0001 as indicated, n=5-6/group).

Supplementary Figure 9 Comprehensive Schematic Illustrating the Integrin-Mediated Signaling Pathway of SEMA7A in intestinal inflammation (Created with BioRender.com).
