# Supplementary figures and images for: Semaphorin 7A is protective during inflammatory peritonitis through integrin receptor signaling
Source: Front Immunol. 2023 Nov 29;14:1251026. doi: 10.3389/fimmu.2023.1251026 (PMC10716335; doi:10.3389/fimmu.2023.1251026)

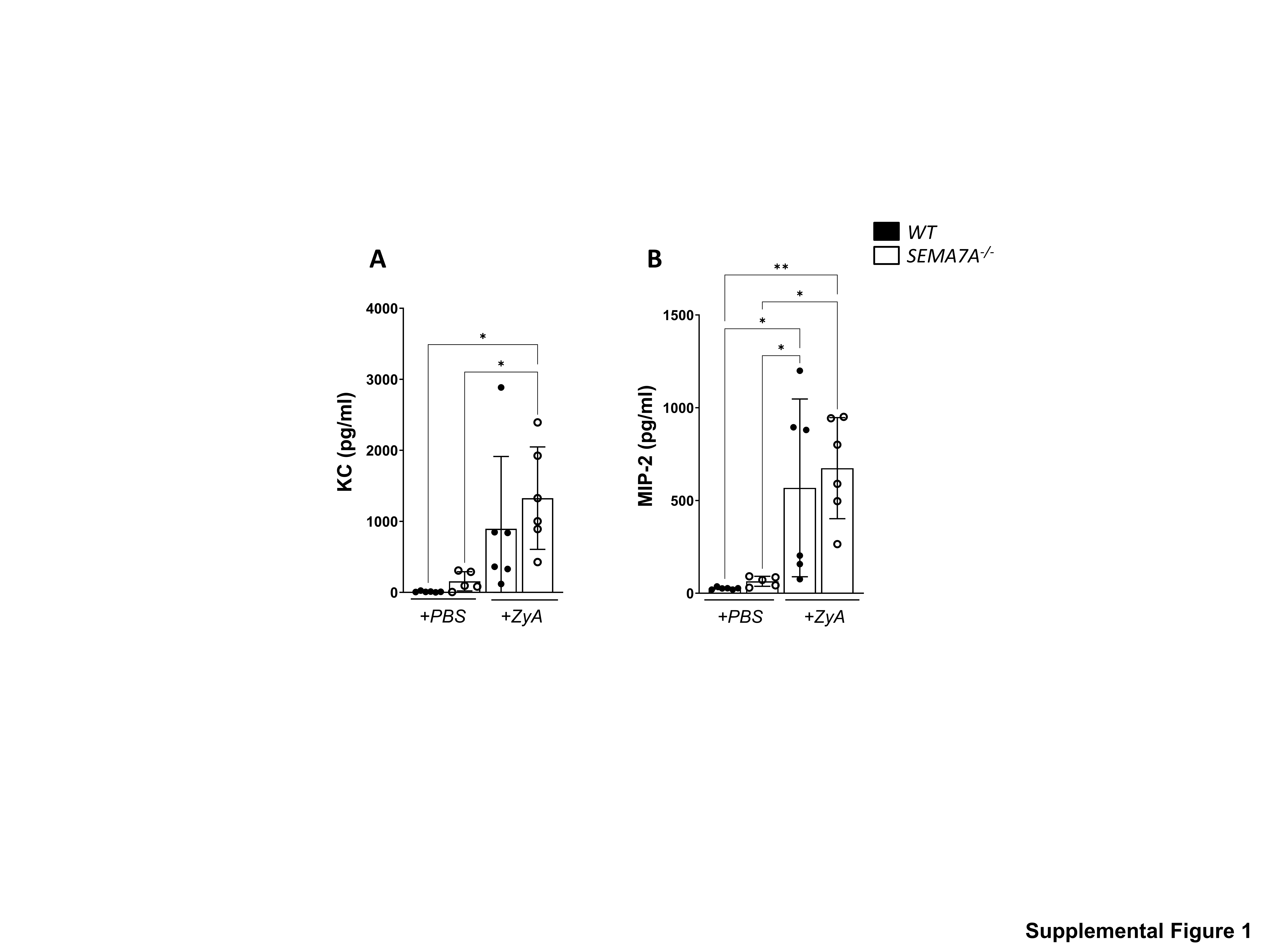

Supplement: Supplementary Figure 1 — Inflammation markers in the peritoneal lavage of WT and SEMA7A-/- animals. Peritonitis was induced in WT and SEMA7A-/- animals through intraperitoneal injection of ZyA. In the control group, PBS-administration was used. The degree of inflammation was determined after 4 hours of incubation by measuring (A) KC and (B) MIP-2 levels (pg/ml) in the removed peritoneal lavage (all data are mean ± SD, *P < 0.05; **P < 0.01 as indicated, n=5-6/group). [file Image_1.tif]

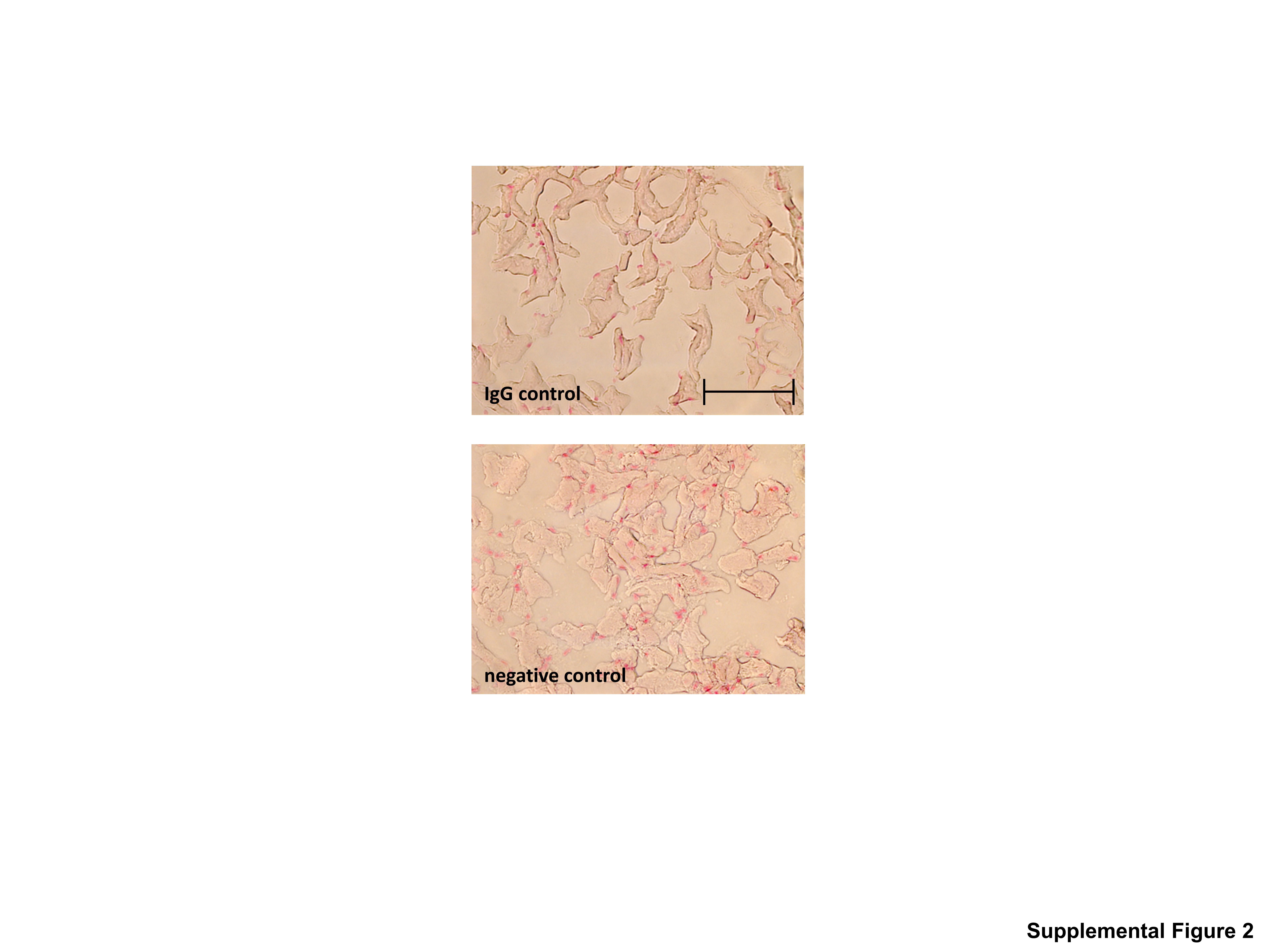

Supplement: Supplementary Figure 2 — IgG and negative controls of PMN staining in histological sections of peritoneum in WT animals after intraperitoneal injection of ZyA (scale bar 100 μm). [file Image_2.tif]

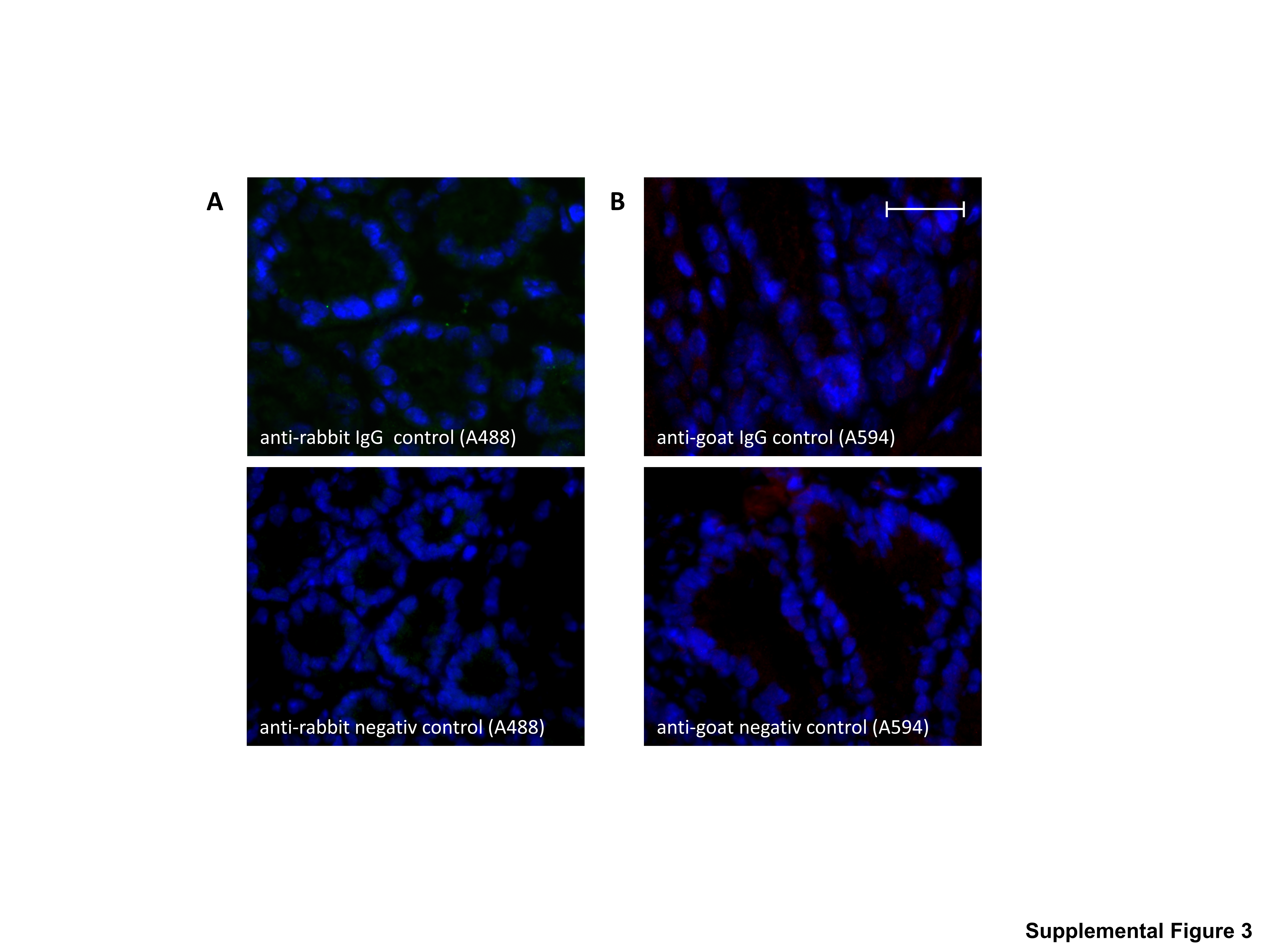

Supplement: Supplementary Figure 3 — Depiction of IgG and negative controls for immunofluorescence staining (A) SEMA7A and (B) Cytokeratin of colon tissue in WT animals (scale bar 50 μm). [file Image_3.tif]

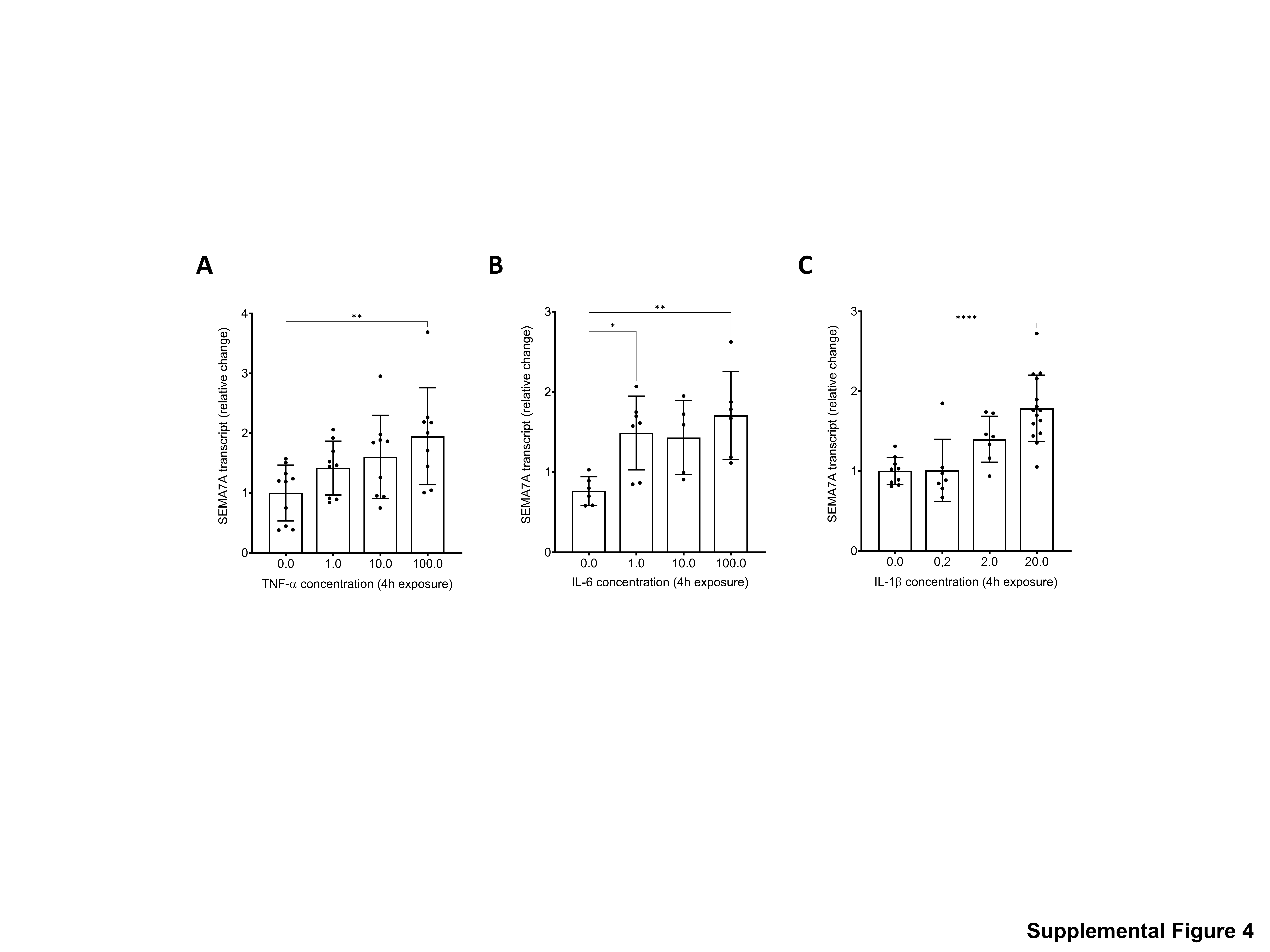

Supplement: Supplementary Figure 4 — Expression of SEMA7A in CaCo-2 through cytokines is dose-dependent. Human intestinal CaCo-2 cells were stimulated with increasing doses of pro-inflammatory cytokines. RT-PCR analysis of SEMA7A-mRNA following (A) increasing concentrations of TNF-α, (B) increasing concentrations of IL-6, and (C) increasing concentrations of IL-1β and incubation for 4 hours (All data are mean ± SD, *P < 0.05; **P < 0.01; ****P < 0.0001 as indicated, n=6-15/group). [file Image_4.tif]

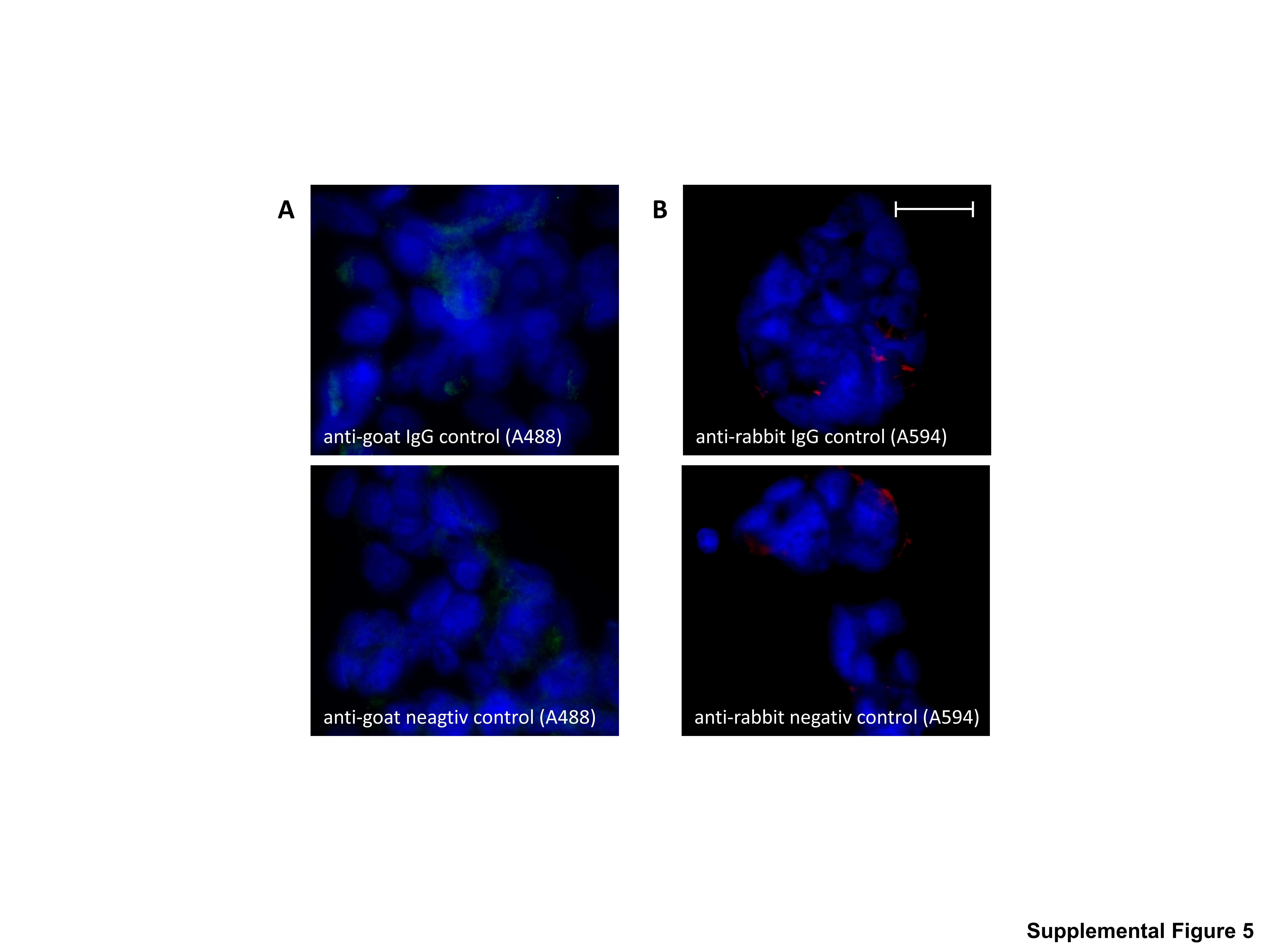

Supplement: Supplementary Figure 5 — Demonstration of the IgG and negative controls of CaCo-2 cell culture staining for (A) SEMA7A and (B) ß-Actin (scale bar 50 μm). [file Image_5.tif]

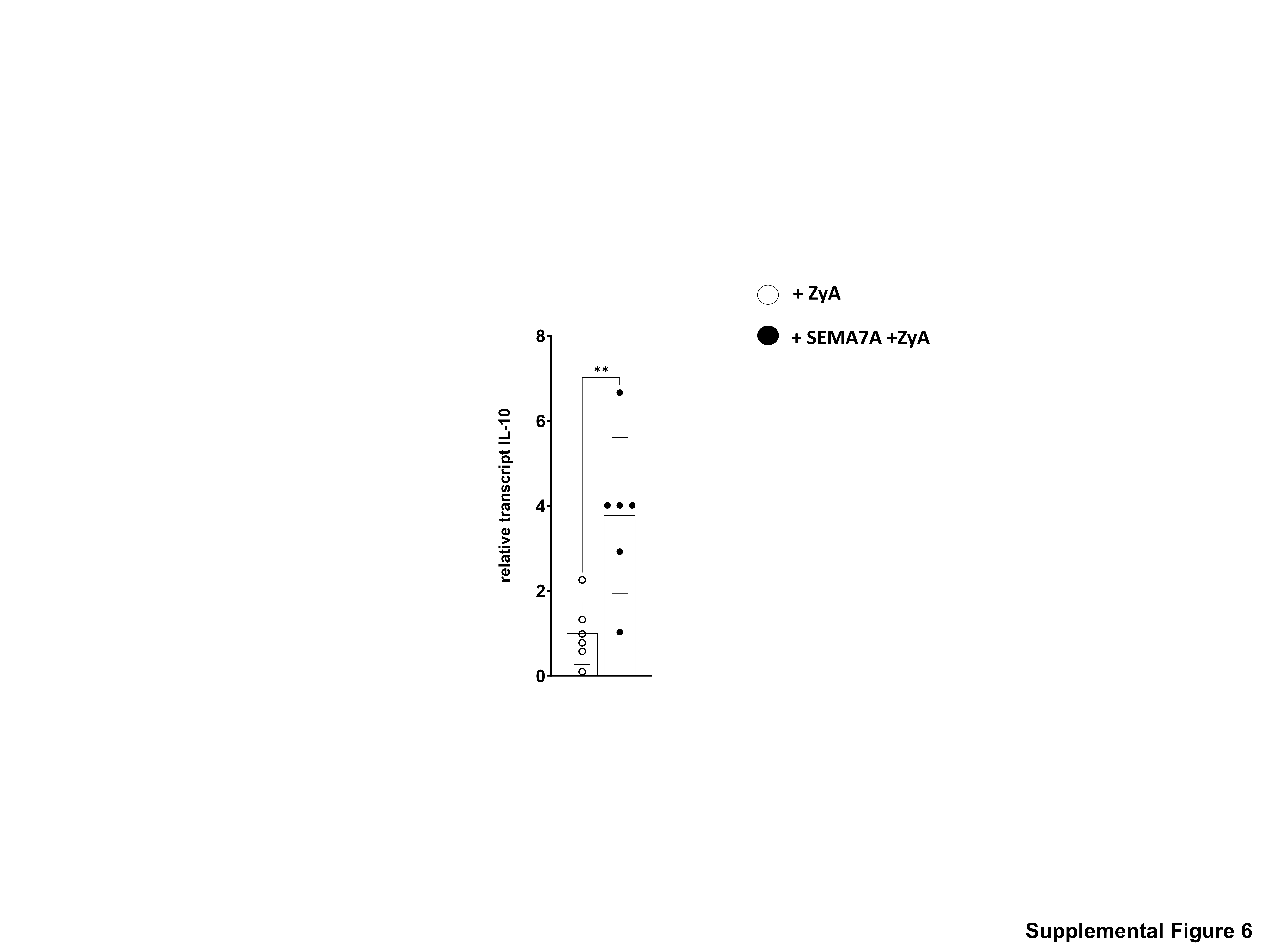

Supplement: Supplementary Figure 6 — Effect of recombinant SEMA7A on the mRNA expression level of IL-10. A co-culture of CaCo-2 and MM6 cells was treated with ZyA (50µg/ml) and SEMA7A (100ng/ml) or ZyA only for 4 hours. The IL-10 expression level was measured by RT-PCR analysis (All data are mean ± SD, **P < 0.01 as indicated, n≥6/group). [file Image_6.tif]

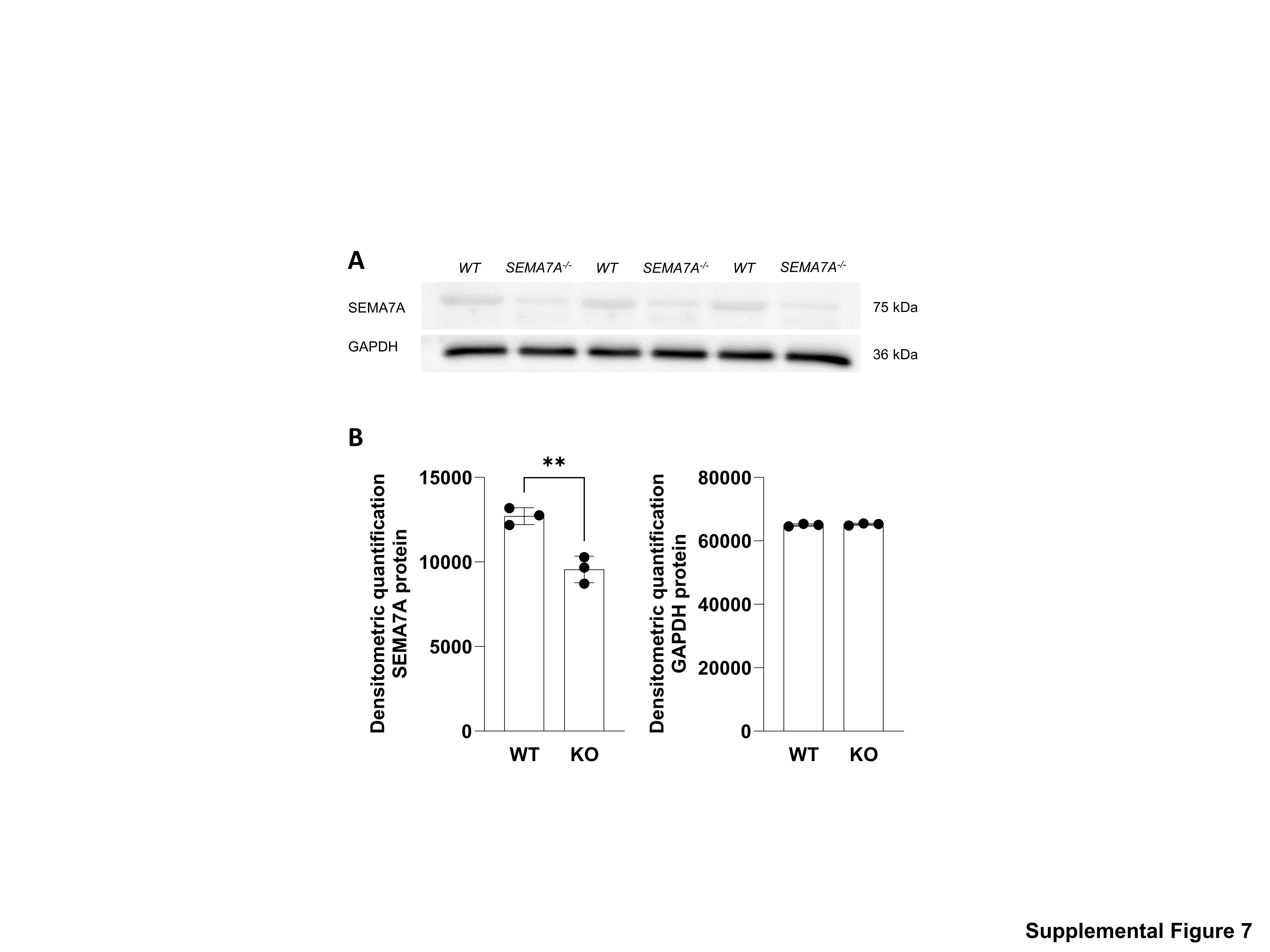

Supplement: Supplementary Figure 7 — Validation of antibody-specificity. Western Blot of Intestinal tissue from Sema7A WT and SEMA7A-/- mice with SEMA7A antibody (A) and densitometric quantification (B) (All data are mean ± SD, **P < 0.01 as indicated, n=3/group). [file Image_7.tif]

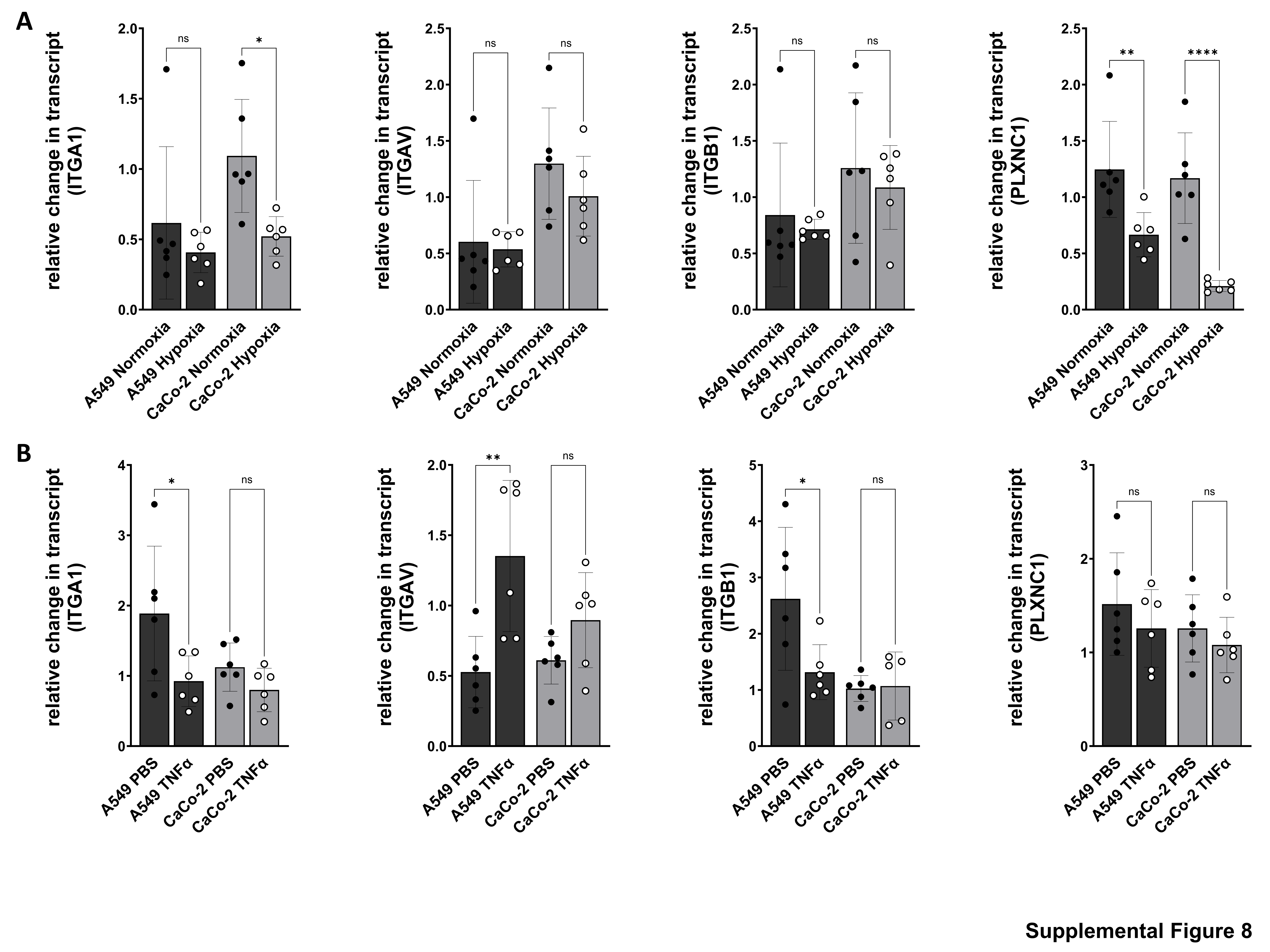

Supplement: Supplementary Figure 8 — mRNA expression levels of A549 and CaCo2-cells upon stimulation with hypoxia or TNFα. CaCo-2 and A549 cells were stimulated with either hypoxia (A) or (B) TNFα and mRNA expression levels of ITGA1, ITGAV, ITGB1, and PLXNC1 were measured by RT-PCR analysis (All data are mean ± SD, ns = not significant *P<0.05, **P < 0.01, ****P<0,0001 as indicated, n=5-6/group). [file Image_8.tif]

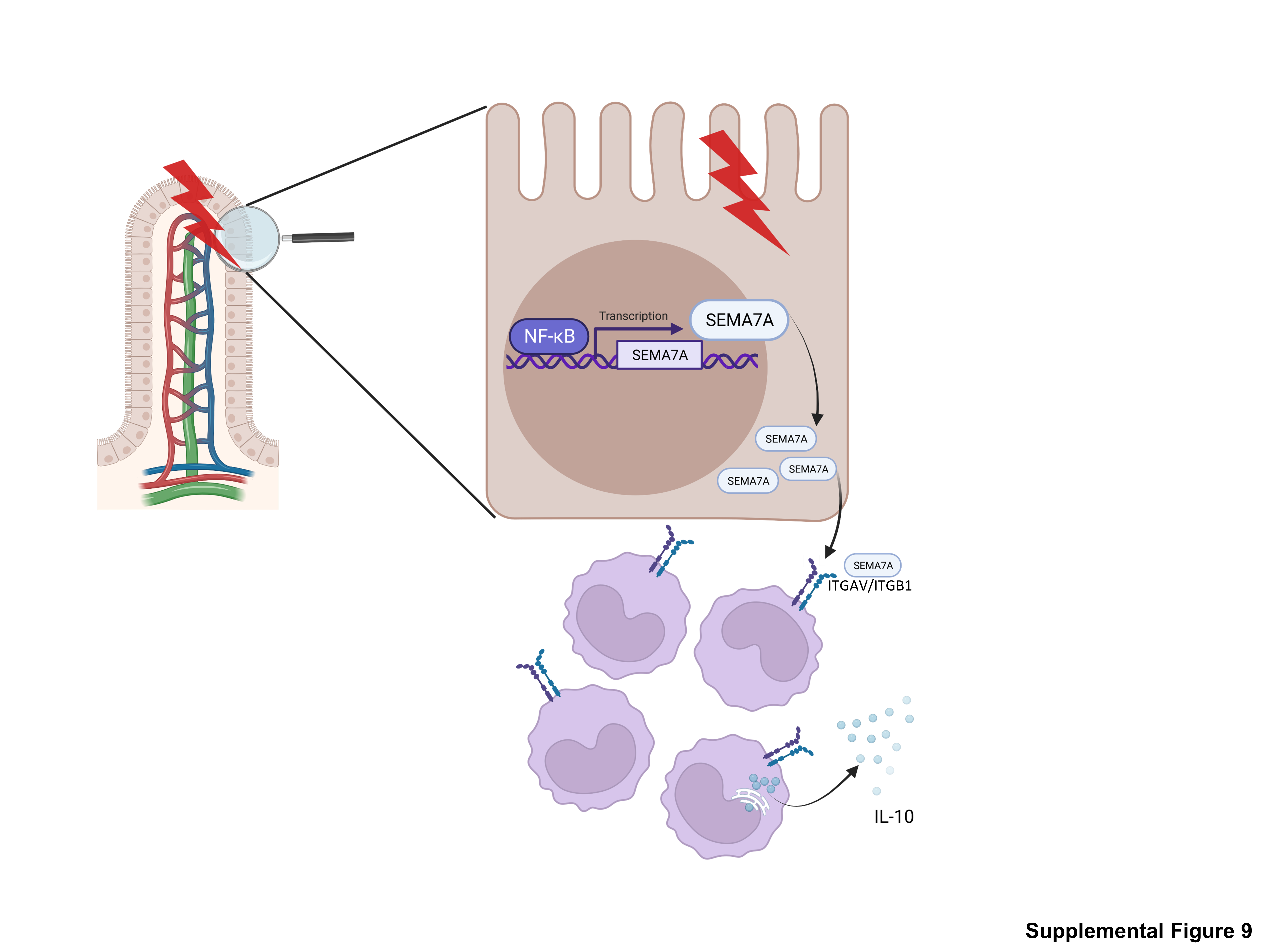

Supplement: Supplementary Figure 9 — Comprehensive Schematic Illustrating the Integrin-Mediated Signaling Pathway of SEMA7A in intestinal inflammation (Created with BioRender.com). [file Image_9.tif]
